# Sputter Deposition of Titanium on Poly(Methyl Methacrylate) Enhances Corneal Biocompatibility

**DOI:** 10.1167/tvst.9.13.41

**Published:** 2020-12-23

**Authors:** Sina Sharifi, Mohammad Mirazul Islam, Hannah Sharifi, Rakibul Islam, Per H. Nilsson, Claes H. Dohlman, Tom Eirik Mollnes, Eleftherios I. Paschalis, James Chodosh

**Affiliations:** 1 Disruptive Technology Laboratory and Schepens Eye Research Institute, Massachusetts Eye and Ear; Department of Ophthalmology, Harvard Medical School, Boston, MA, USA; 2 Department of Immunology, Oslo University Hospital, Rikshospitalet, University of Oslo, Oslo, Norway; 3 Linnaeus Center for Biomaterials Chemistry, Linnaeus University, Kalmar, Sweden; 4 Research Laboratory, Nordland Hospital, Bodø, Norway; Faculty of Health Sciences, K.G. Jebsen TREC, University of Tromsø, Tromsø, Norway; 5 Centre of Molecular Inflammation Research, Norwegian University of Science and Technology, Trondheim, Norway

**Keywords:** poly(methyl methacrylate) (PMMA), titanium sputtered coating, Boston Keratoprosthesis, artificial cornea, biocompatibility

## Abstract

**Purpose:**

To evaluate titanium (Ti) sputtering of the poly(methyl methacrylate) (PMMA) stem of the Boston Keratoprosthesis (BK) as a method to enhance interfacial adhesion between the PMMA and the recipient corneal tissue.

**Methods:**

PMMA specimens were plasma treated with Ar/O_2_ and coated with Ti using a DC magnetron sputtering instrument. The topography and hydrophilicity of the surfaces were characterized using atomic force microscopy and a water contact angle instrument, respectively. Scratch hardness and adhesion of the Ti film were measured using a mechanical tester. Biocompatibility assessments were performed using cultured human corneal fibroblasts and whole blood ex vivo. The optical quality of the Ti sputtered BK was evaluated using a custom-made optical bench.

**Results:**

By contact angle studies, the Ti coating improved PMMA hydrophilicity to match that of medical-grade Ti (Ti-6Al-4V-ELI). Ti sputtering of contact surfaces resulted in a plate-like morphology with increased surface roughness, without impacting the transparency of the BK optical component. Scratch testing indicated that the mechanical behavior of the Ti coating was similar to that of casted Ti, and the coating was stable in pull-off adhesion testing. Sputtered Ti film was highly biocompatible based on tests of cell viability, adhesion, proliferation, differentiation, collagen deposition, and keratocan expression, the properties of which exceeded those of uncoated PMMA and did not induce increased complement activation.

**Conclusions:**

Titanium coating of the BK stem generated a mechanically and biologically favorable interface, which may help to enhance corneal stromal adhesion and biocompatibility.

**Translational Relevance:**

Improving the biocompatibility of the BK PMMA stem may improve long-term outcomes of implantation.

## Introduction

The Boston Keratoprosthesis (BK) is a medical device indicated for patients with corneal blindness not amendable to standard corneal transplantation.^1^^–^^12^ The BK is composed of a front plate/stem (optical portion) made of poly(methyl methacrylate) (PMMA) and a back plate made of PMMA or titanium (Ti), assembled in bolt (PMMA front plate/stem) and nut (Ti back plate) fashion within a donor cornea that serves as a carrier; the donor cornea is sutured to the eye as in standard corneal transplantation.^13^^–^^15^ The BK is globally the most commonly implanted artificial cornea. However, poor interfacial adhesion between the PMMA stem and the recipient corneal tissue may adversely affect clinical outcomes after implantation.^16^ To improve the interfacial adhesion, numerous surface modification approaches have been proposed, including biomineralization of hydroxyapatite (HAp) onto the PMMA surface,^17^ calcium phosphate coating (d-CaP),^18^ TiO_2_ nanoparticle dip coating,^19^ TiO_2_ coating over a layer of polydopamine,^20^ covalent functionalization with l-3,4-dihydroxyphenylalanine (l-DOPA),^21^ and nanopatterning with cell adhesive peptides.^22^

**Figure 1.**
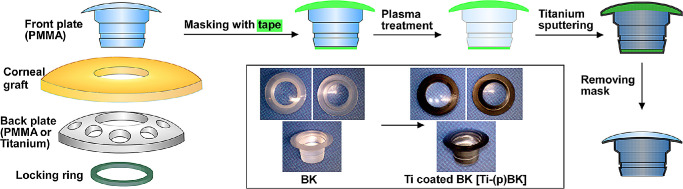
Illustrations of core–skirt architecture of a BK and the Ti sputtering process to form a Ti film on the BK.

The superiority of Ti compared to PMMA as a substrate for cell growth has been previously demonstrated.^14^^,^^15^^,^^23^^,^^24^ The application of a sandblasted Ti sleeve around the BK optic was previously shown to enhance the adhesion between the sleeve and a donor corneal graft^20^; yet, it is expensive and requires precise machining of the Ti sleeve to perfectly fit the stem. Herein, we investigate the use of magnetron sputtering for nanometer-thickness Ti deposition on the PMMA stem of the BK (Fig. 1). We demonstrate uniform Ti film deposition and good adherence to the PMMA substrate with favorable mechanical properties. The Ti surface coating improved biocompatibility in vitro and may help improve long-term device retention and performance in vivo.

## Materials and Methods

### Sample Preparation

Medical-grade PMMA (Rod number 2; PolyOne Corporation, Littleton, MA) discs with 0.5-mm thickness and 40.0-mm diameter were cut to 8.0-mm diameter by a laser cutter (Helix 75 Watt CO_2_ Laser Engraver; Epilog, Golden, CO), washed extensively with detergent and water, and dried under ambient conditions. Plasma treatment was carried out using Femto Plasma Cleaner (Diener Electronic GmbH + Co., Ebhausen, Germany) at 100 W for 2 minutes under an argon/oxygen 80/20 mixture to produce plasma-treated PMMA, or (p)PMMA. Immediately after surface treatment, PMMA discs or the BK with the stems facing down (anterior down) were taped to the sample holder using Kapton tape, and a thin Ti film was deposited on the PMMA substrates, PMMA and (p)PMMA, using a DC magnetron sputtering instrument (Orion 3; AJA International, Inc., North Scituate, MA) to generate Ti-coated PMMA (Ti-PMMA) and plasma-treated then Ti-coated PMMA (Ti-(p)PMMA), respectively. The sputtering chamber was pumped to a vacuum level below 2.0 × 10^–6^ Torr prior to coating. During the deposition, the sample holder was rotated at 40 rpm. The distance between the substrate holder and the sputtering target (Ti with a purity of 99.999%) was set at 15.0 cm. Deposition was conducted under 200-W DC power with pressure of 4 × 10^–3^ Torr with argon gas for 2 hours at a temperature of 40°C. To protect the optical components, the front and back (optical surfaces) were covered with 5- and 2.5-mm-diameter tapes, before plasma treatment, to be removed after Ti deposition. The thickness of Ti layer was measured by a profilometer (Dektak 6M Profilometer; Veeco, Plainview, NY) by assessing the height difference between the deposited layer and the substrate on the PMMA discs from multiple points (*n* = 8), with the exclusion of the wall-type features of the Ti film. Titanium discs of 15.6-mm diameter were prepared using Ti-6Al-4V-ELI medical-grade Ti (grade 23) of 580-nm thickness, cleaned and polished prior to use.

### Atomic Force Microscopy

The topography of the surface of the PMMA discs was probed using an Asylum Research Cypher atomic force microscope (AFM; Asylum Research, Oxford Instruments, High Wycombe, UK) in AC mode using a standard AFM probe (Opus 160AC-NA; MikroMasch, Sofia, Bulgaria). Topographical surface data were acquired in phase and height profiles with scan sizes of 30 × 30 and 2 × 2 µm^2^ at a rate of 2.0 Hz. The set point and the drive amplitude were adjusted at 775 and 266 mV, respectively. Surface roughness was calculated based on a 30 × 30-µm^2^ scan size (*n* = 8 per group).

### Water Contact Angle Measurement

Surface hydrophilicity of the Ti coating on the PMMA discs was assessed with a contact angle and surface tension measurement system (FTA100; First Ten Angstroms, Inc., Newark, CA) using a static sessile drop technique. At room temperature, a 5-µL droplet of distilled water was deposited by a syringe located above the sample surface, and the image was captured from the side by a high-resolution camera. The contact angle for each group was analyzed using FTA 2.1 software and averaged for each group (*n* = 8 per group).

### X-Ray Photoelectron Spectroscopy

X-ray photoelectron spectroscopy (XPS) spectra of the PMMA discs (before and after sputtering) were acquired in the range from 100 to 1300 eV using a Thermo Scientific K-Alpha spectrometer (Thermo Fisher Scientific, Waltham, MA). Spectra were obtained via Avantage software (Thermo Fisher Scientific) in the survey and high-resolution modes with an energy step size of 1 eV and 10 scans.

### Optical Evaluation

Transparency-resolution studies of the BK before and after Ti coating were carried out using an optical bench, as previously described.^25^ Untreated and Ti-(p)PMMA BKs (*n* = 7) were individually held in a diaphragm in front of the microscope located 304.8 cm away from an illuminated 1951 USAF resolution test chart (Edmund Optics, Barrington, NJ).^26^ Three independent observers, masked to the treatment groups, observed the bar chart via the microscope to determine the smallest identifiable three-bar feature on the chart. The resolution was calculated and averaged for each observer and then averaged for the groups (Ti-coated and non-coated BKs) according to the following equation, as previously described^25^:

$$\begin{array}{c} Resolution\;\left(\mathrm{lp}/\mathrm{mm}\right)=2^{\left(Group\;number+\frac{Element\;number-1}{6}\right)} \end{array}$$

The imputed visual acuity was extracted after converting the element numbers of the resolution chart into their corresponding optotypes, based on distance and angle of the optotype bars as previously described.^25^ For example, Element Number 2 in Group Number −1 would correspond to a visual acuity of 20/20 at 20 feet. The independent values obtained from the three observers were averaged to find the visual resolution for each analyzed BK (*n* = 8 per group).

### Mechanical Evaluation

#### Coating Adhesion Test

The adhesion strength between the Ti coating and the PMMA discs was tested with the pull-off method using a mechanical tester (ESM303; Mark-10, Copiague, NY) according to the ASTM D4541-17 standard.^27^ The circular stud used had a diameter of 2 mm (area, 3.14 mm^2^). Surface contaminants were washed off the stud using a detergent solution followed by acetone. The stud was adhered to an 8-mm-diameter sample or PMMA, Ti-PMMA, or Ti-(p)PMMA with a thermally curable epoxy adhesive (Loctite Hysol 0151 Epoxy Adhesive; Henkel Corporation, Rocky Hill, CT) and cured at 50°C for 6 hours; in the stability study, the samples were soaked in Dulbecco's Modified Eagle Medium: Nutrient Mixture F-12 (DMEM/F-12), 1:1 (Corning, Inc., Corning, NY), for a week before the measurement. The tensile strength of the Ti coating was assessed by measuring the mechanical force required to separate the Ti film from the substrate (*n* = 8 per group).

#### Scratch Test

The scratch resistance of Ti-coated PMMA discs was assessed with increasing normal load^28^ using the ESM303 mechanical tester equipped with a Newport XY stage (423 series; Newport Corporation, Irvine, CA) and sharp steel stylus with a spherical apex diameter of 0.05 mm. Specimens were attached to a glass slide, which was tilted at 0.9°. A stylus was gradually lowered to the surface of the specimen, and the sample was linearly moved, resulting in a gradually increasing normal load with the load proportional to the sliding distance. The scratch test was stopped at the moment when delamination of the coating was observed with a high-resolution camera (Dino-Lite Edge AM73915MZTL, 5MP; Dino-Lite, Torrance, CA), and the load at that point was recorded using Mark-10 MESURgauge Plus software (*n* = 8 per group). In the stability study, the samples were soaked in cell culture media (DMEM/F-12 media supplemented with 10% fetal bovine serum; Thermo Fisher Scientific)^25^ and incubated at 37°C and 5% CO_2_ for 1 week before measurement.

### In Vitro Biocompatibility

#### Live/Dead Assay

To evaluate biocompatibility, 8.0-mm-diameter Ti-(p)PMMA and PMMA discs were fitted to 48-cell culture well plates in 1.0-mL penicillin/streptomycin 5× solution for 15 minutes and then washed with sterile PBS. Then, 5000 human corneal fibroblasts (HCFs), cultured from deceased human corneal donors, were seeded on each disc in 500 µL of DMEM/F-12 media supplemented with 10% fetal bovine serum^25^ and incubated at 37°C and 5% CO_2_ for up to 7 days. The cell culture media were changed every other day. After 1, 4, and 7 days of cell culture, live/dead staining was performed using an Invitrogen LIVE/DEAD staining kit (Thermo Fisher Scientific) composed of calcein acetoxymethyl and ethidium homodimer-1, and imaging was performed using an inverted fluorescent microscope (Zeiss Axio Observer Z1; Carl Zeiss Microscopy GmbH, Jena, Germany) with 10× objective. Four samples per group were tested and compared to those of tissue culture well plate (TCP) and medical-grade casted Ti discs as positive controls. Four images obtained from each sample were analyzed by ImageJ software (National Institutes of Health, Bethesda, MD) to assess cell viability and confluency as described elsewhere.^29^^–^^31^

#### alamarBlue Assay

The metabolic activity of HCFs cultured on Ti-(p)PMMA discs was assessed using the Invitrogen alamarBlue assay (Thermo Fisher Scientific). HCF cells were cultured on the discs as above. The alamarBlue assay was performed at days 1, 4, and 7 days of culture using fresh culture media containing 0.004% w/v resazurin sodium salt (Sigma-Aldrich, St. Louis, MO) in 500 µL and incubated for 2 hours at 37°C. Next, 300 µL of reacted media was transferred to a new 96-well plate (100 µL in each well) and read on a Synergy 2 plate reader (BioTek Instruments, Winooski, VT) at 600/25 nm, normalized with the cell-free media. Four samples per group were tested and compared to those of TCP and casted Ti disc controls.

#### Immunocytochemistry

The expression of aldehyde dehydrogenase (ALDH3A1), integrin β1, α-smooth muscle actin (α-SMA), collagen I, and keratocan by HCFs cultured on Ti-(p)PMMA was determined by fluorescence immunocytochemistry after 6 or 45 days of cell culture. At the end of each culture period, the discs were removed from the media, rinsed with PBS, and fixed in 4% paraformaldehyde. Cells were permeabilized with 0.25% Triton X-100 and blocked with 5% fetal bovine serum in 0.05% Tween-20 in PBS. Cells were incubated with the following primary antibodies overnight at 4°C in humidified conditions: (1) mouse monoclonal antibody against ALDH3A1 (clone 1B6, GTX84889, dilution 1:100; GeneTex, Irvine, CA); (2) rabbit polyclonal antibody against integrin β1 (GTX112971, dilution 1:250; GeneTex); (3) mouse monoclonal antibody against α-SMA (clone 1A4, ab7817, dilution 1:200; Abcam, Cambridge, UK); (4) mouse monoclonal antibody against collagen I (COL-1, ab90395, dilution 1:500; Abcam); and (5) goat polyclonal antibody against keratocan (C-16, sc-33243, dilution 1:100; Santa Cruz Biotechnology, Inc., Dallas, TX). The specimens were incubated with specific secondary antibodies—Goat Anti-Mouse IgG H&L (FITC) (ab6785, dilution 1:1000; Abcam) or Goat Anti-Rabbit IgG H&L (FITC) (ab6717, dilution 1:1000; Abcam)—for 1 hour at room temperature, washed with PBS, and mounted in VectaShield mounting media containing 4′,6-diamidino-2-phenylindole (DAPI; Vector Laboratories, Burlingame, CA). They were then imaged using the Zeiss Axio Observer Z1 inverted fluorescent microscope.

#### Total Collagen Quantification

HCFs were cultured as above, and after 45 days the total amount of collagen deposition per group (PMMA, Ti-(p)PMMA, and Ti) was quantified using a total collagen assay kit (MAK008; Sigma-Aldrich Corp.) according to the manufacturer's protocol.^32^^,^^33^ Briefly, the cell-secreted collagen matrix was removed from each specimen, homogenized with 1 mL of HCl (6 M) and hydrolyzed in a 95°C dry bath overnight. Samples were placed in wells of a 96-well plate, and the plates were baked at 60°C until dry. Residues were then incubated with chromogenic assay reagents at 60°C for 90 minutes. Total collagen quantification was performed by measuring the absorbance of reacted solution at 560 nm using a microplate reader. Serial-diluted hydroxyproline was used as standard. All samples and standards were performed in triplicate.

#### Ex Vivo Complement Activation

Human blood was drawn from healthy volunteers into Vacutainer tubes (Becton, Dickinson and Company, Plymouth, UK) containing a specific thrombin inhibitor, lepirudin (Refludan; Aventis Pharma, Mumbai, India), at a final concentration of 50 µg/mL. All experimental protocols were approved by the ethics committee at Oslo University Hospital (REK SØR S-04114), and the all methods were carried out in accordance with relevant guidelines and regulations, conforming to the tenets of the Declaration of Helsinki. Informed written consent was obtained from each donor.

For each set of experiments, 300 µL of the blood was aliquoted into four 1.8-mL round-bottom sterile polypropylene cryogenic vials (Thermo Fisher Scientific). In one vial, ethylenediaminetetraacetic acid (EDTA) was added immediately at a 10-mM final concentration, thereby stopping further complement activation, in order to measure the complement activation status at time zero (T0). The other three vials containing whole blood were incubated at 37°C as follows. PMMA discs (8-mm diameter) were placed in one vial and Ti-(p)PMMA discs in another. The third vial contained only whole blood. After incubation, EDTA was added to stop further complement activation, and plasma was separated for preservation. The incubated whole blood without an immersed specimen served as a negative control for complement activation. Three sets of experiments were carried out with samples collected after 60, 90, and 120 minutes of incubation. Five independent sets of experiments were performed at 30 minutes of incubation. Collected plasma was preserved at –70°C. Complement activation was assessed by measuring soluble C3bc fragments and the soluble terminal complement complex (TCC) sC5b-9 and using enzyme-linked immunosorbent assays as described previously.^34^ C3bc was determined using monoclonal antibody bH6 for capture and polyclonal rabbit anti-C3c (Behringwerke AG, Marburg, Germany) and peroxidase-labeled anti-rabbit immunoglobulin (GE Healthcare, Chicago, IL) as detection antibodies. Levels of sC5b-9 were determined with anti-neo C9 monoclonal antibody aE11 for capture^35^ and biotinylated monoclonal anti-C6 (clone 9C4) as previously described.^34^^,^^35^ Streptavidin–HRP conjugate (GE Healthcare) was added for detection.

### Statistical Analysis

One-way analysis of variance (ANOVA) with Tukey comparison test was used to compare surface roughness, contact angle, adhesion strength, scratch hardness, viability, metabolic activity, collagen content, and TCC among groups. Student's *t*-test (two-tailed) was used to compare resolution and imputed visual acuity between groups (Ti-coated and non-coated BKs). A value of *P* < 0.05 was considered statistically significant. Prism 8.3.0 (GraphPad, San Diego, CA) was used to analyze the data.

## Results

### Titanium Coating Characterization

#### Surface Topography and Film Thickness

**Figure 2.**
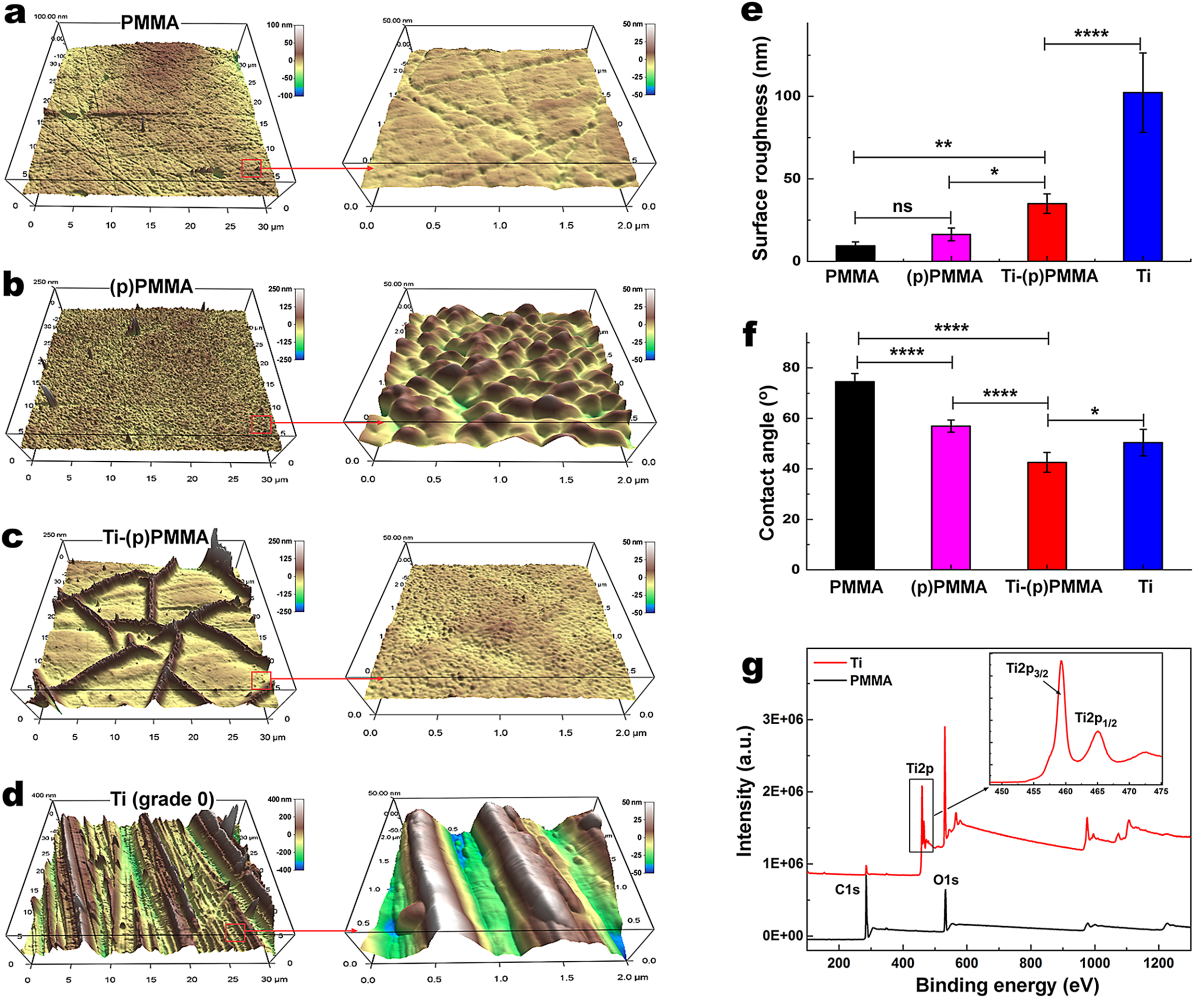
Morphological characterization of PMMA surface before plasma treatment (**a**), after plasma treatment (**b**), and after subsequent Ti sputtering (**c**) compared to Ti (**d**). Two scan sizes (30 × 30 and 2 × 2 µm^2^) are shown. Surface roughness (**e**) was analyzed using AFM. (**f**) Contact angle values of PMMA surfaces after Ti sputtering as compared to Ti; morphological studies indicate that the sputtering altered the topography and contact angles of the PMMA samples. ^*^P < 0.05, ^**^P < 0.01, ^***^P < 0.001, and ^****^P < 0.0001; ns, *P* > 0.05. (**g**) XPS characterization of the PMMA before and after Ti sputtering in survey spectra along with the high-resolution spectra (*inset*). ns, not significant.

The nanoscale surface topography of specimens of PMMA, (p)PMMA, and Ti-(p)PMMA, with casted Ti serving as a control, was studied by AFM (Figs. 2a–e). AFM showed that all surfaces had different morphological properties. PMMA showed relatively low surface roughness (root mean square [RMS] = 9.34 ± 2.39 nm (Figs. 2a, 2b). (p)PMMA samples showed increased roughness with extensive and homogeneous repeats of surface peaks (RMS = 16.29 ± 3.85 nm) (Figs. 2a, 2b). Ti-(p)PMMA surfaces showed a plate-like morphology, with the Ti plates forming junctions protruding from the surface and creating wall-type futures with a significant increase in surface roughness (RMS = 34.92 ± 5.85 nm) compared to PMMA and (p)PMMA (*P* = 0.00002 and *P* = 0.00034, respectively). Compared to all other groups, casted Ti showed an even higher roughness with parallel grooves (RMS = 102.22 ± 24.07 nm) (Fig. 2d). The thickness of the Ti layer was assessed by a profilometer. The analysis indicated deposition of a Ti film 550 ± 50 nm thick on the PMMA after 2 hours of sputtering.

#### Water Contact Angle

The Ti coating surface energy and hydrophilicity were assessed using water contact angle measurements. The contact angles for PMMA, (p)PMMA, and Ti-(p)PMMA were 74.5° ± 3.2°, 56.9° ± 2.4°, and 42.6° ± 3.9°, respectively, as compared to casted Ti (50.4° ± 5.2°) (Fig. 2f). ANOVA analysis showed pairwise statistical differences (*P* < 0.05) among all groups.

#### Chemical Characterization

XPS spectra of PMMA discs (PMMA and Ti-(p)PMMA) are shown in Figure 2g. There are two prominent binding energy peaks in the PMMA sample, in addition to C1s and O1s, which are located at 285 eV and 530 eV, respectively. After sputtering, XPS spectra revealed Ti energy binding peaks at 459, 465, and 472 eV, in addition to the binding energy peaks of C1s and O1s, which originated from the underlying PMMA substrate.

### Optical Evaluation

**Figure 3.**
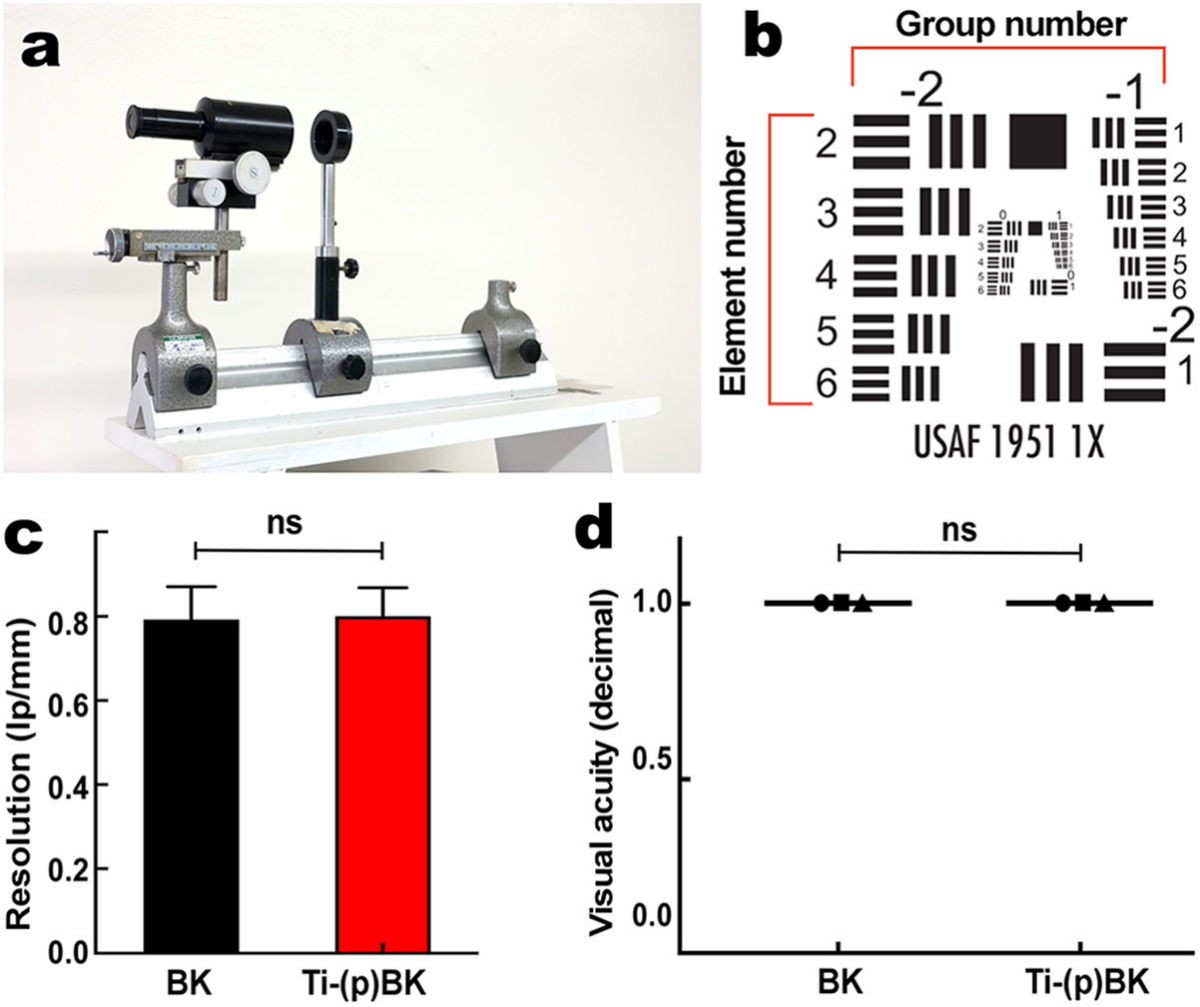
Transparency-resolution analysis using an optical bench composed of a microscope, a diaphragm to hold the BK (**a**) and a resolution chart (**b**) placed 304.8 cm away from the diaphragm. (**c**) The resolution calculation revealed no significant differences between BKs before and after processing (*P* > 0.05). (**d**) Three independent observers found equal sharpness through BKs before and after processing; *P* > 0.05.

Transparency-resolution testing using an optical bench (Fig. 3a) showed no significant differences in resolution among the BK devices before or after processing (Figs. 3a–3c). Resolution measurements before and after processing of BKs were 0.800 ± 0.067 line pairs (lp)/mm and 0.797 ± 0.079 lp/mm, respectively (*P* = 0.94) (Fig. 3c). All three observers could resolve the equivalent of 20/20 visual acuity through all devices before and after processing (Figs. 3a, 3d).

### Mechanical Evaluation

#### Coating Adhesion Test

**Figure 4.**
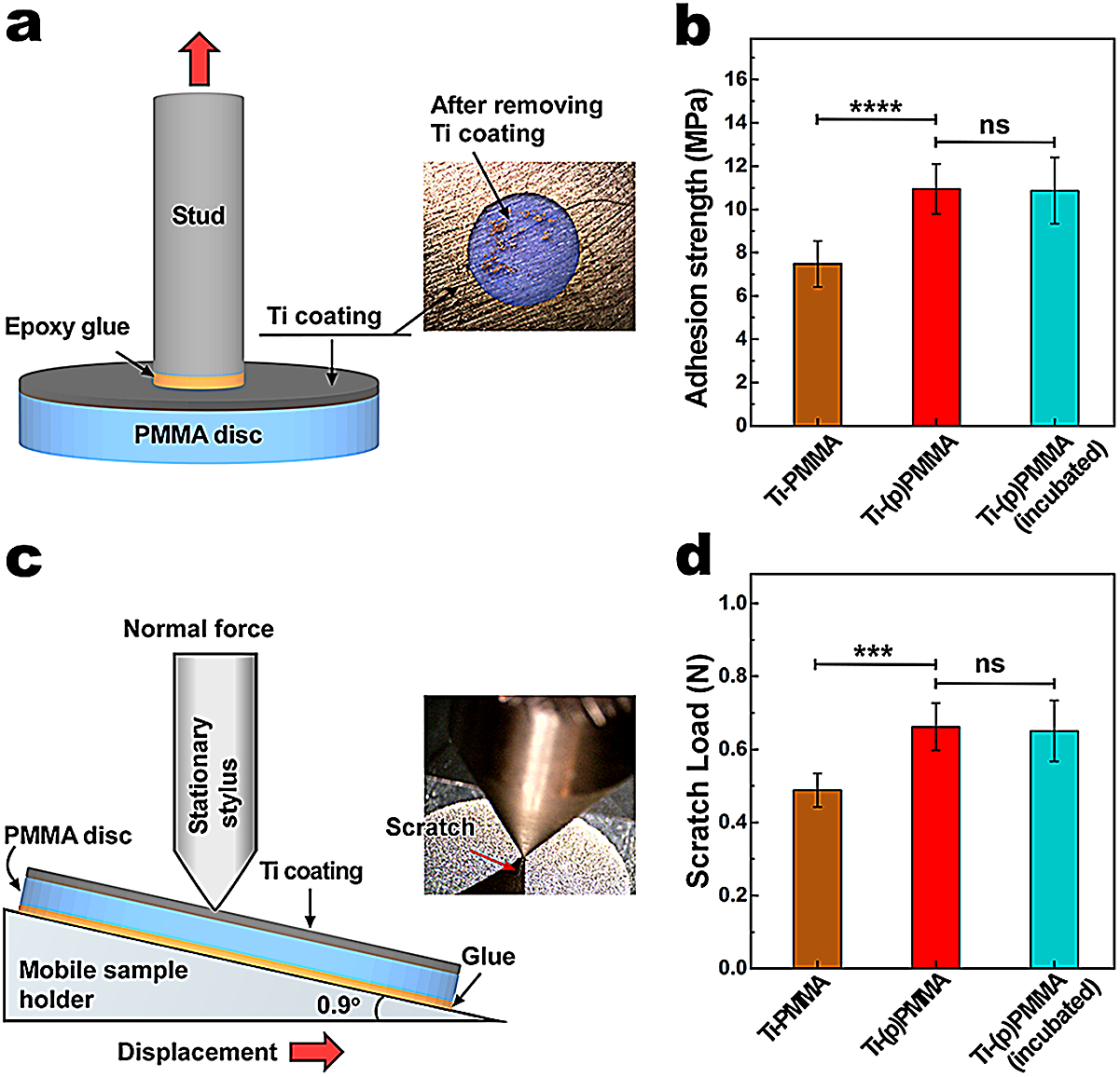
Schematic illustration of adhesion test (**a**) and adhesion strength (**b**) values between sputtered Ti and the underlying PMMA. Plasma-treated and then Ti sputtered film showed significantly higher adhesion to PMMA compared to untreated PMMA. Schematic illustration of scratch test (**c**) with increasing normal load and scratch loads (**d**) for Ti films on PMMA compared to those on (p)PMMA. Plasma-treated and then Ti sputtered film has significantly higher scratch load compared to non-treated PMMA. Incubation in the media for a week also did not alter the adhesion strength and scratch load of Ti film. ^*^P < 0.05, ^**^P < 0.01, ^***^P < 0.001, and ^****^P < 0.0001; ns, *P* > 0.05.

The adhesion of sputtered Ti to PMMA was evaluated using a pull-off adhesion test.^36^ In some samples, the epoxy glue detached from the Ti film, and these were not included in the analyses. Ti adhesion strength on (p)PMMA (10.9 ± 1.1 MPa) was significantly higher than that of the Ti sputtered coating onto untreated PMMA (7.4 ± 1.1 MPa; *P* = 0.00002), as illustrated in Figures 4a and 4b. In addition, Ti adhesion strength on (p)PMMA samples before incubation (10.9 ± 1.1 MPa) was similar to that after incubation in the cell culture media for 1 week (10.8 ± 1.5 MPa; *P* = 0.88).

#### Scratch Test

The scratch load of Ti-(p)PMMA was significantly higher than that of Ti-PMMA (0.488 ± 0.046 N; *P* = 0.001) as shown in Figures 2c and 2d. Ti-(p)PMMA after incubation in the cell culture media for a week showed a similar scratch load (0.651 ± 0.084 N; *P* = 0.998).

### In Vitro Biocompatibility

**Figure 5.**
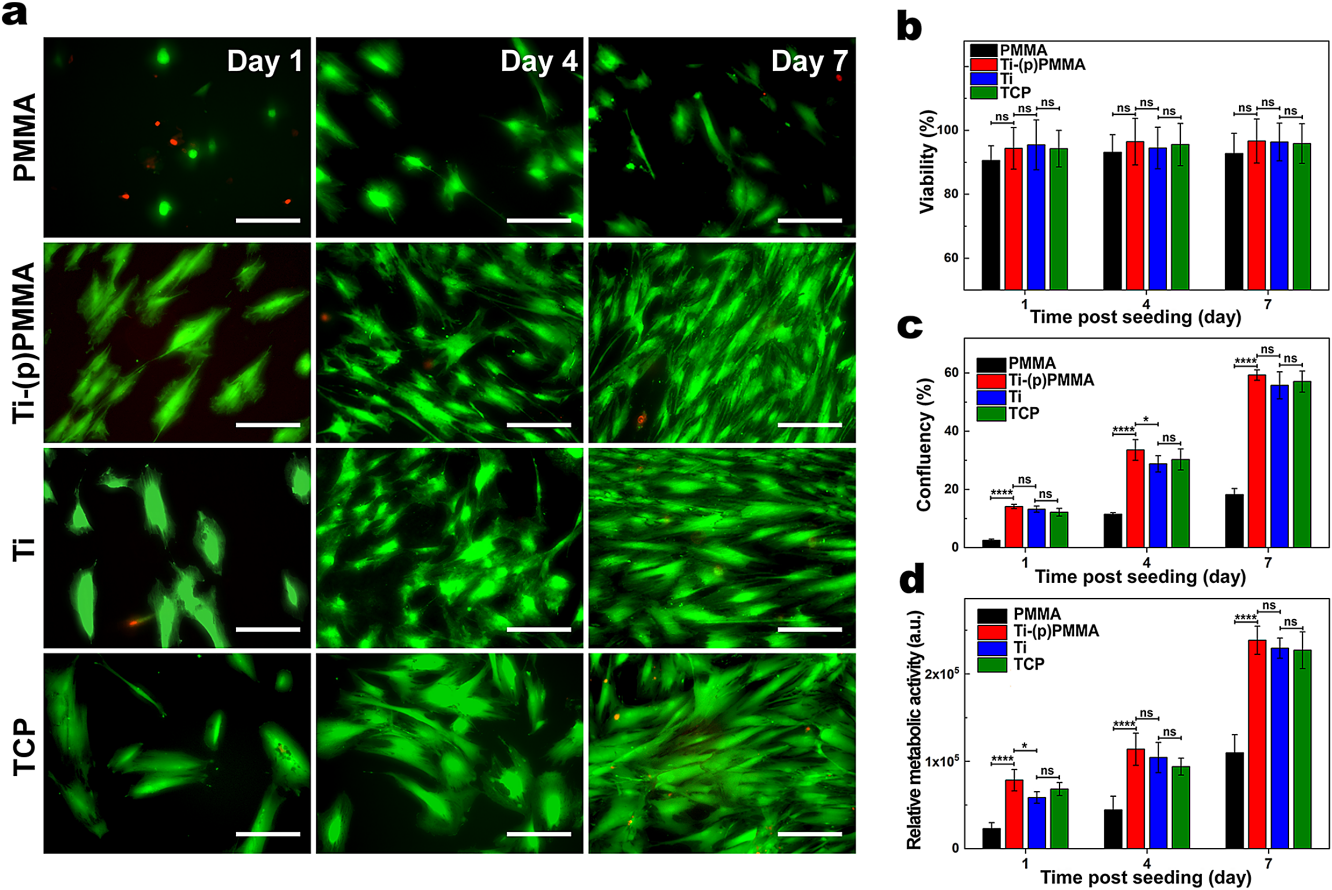
Biocompatibility characterization of functionalized PMMA. Representative LIVE/DEAD images (**a**) of HCFs cultured on PMMA, plasma-treated, and then Ti-(p)PMMA compared to those cultured on Ti or TCPs and their corresponding cell viabilities (**b**) and confluencies (**c**) after 1, 4, and 7 days of cell culture. *Scale* *bar*: 200 µm. Cell viability and confluency were quantified from LIVE/DEAD images using ImageJ software (*green*, calcein AM, live cells; *red*, ethidium homodimer-1, dead cells). (**d**) Quantification of the metabolic activity of HCFs using the alamarBlue assay after 1, 4, and 7 days of cell culture. The cells seeded on Ti-(p)PMMA demonstrated significantly higher fluorescence intensities (higher metabolic activity) at all time points compared to those of PMMA. ^*^P < 0.05, ^**^P < 0.01, ^***^P < 0.001, and ^****^P < 0.0001; ns, *P* > 0.05.

HCFs seeded on all surfaces showed similar cellular viabilities (>90%); however, those seeded on the Ti-(p)PMMA surface exhibited greater confluency (59.3% ± 1.8%) compared to PMMA (18.2% ± 2.1%) after 7 days of cell culture. HCFs cultured on Ti-(p)PMMA demonstrated cell confluency similar to that of casted Ti and TCP controls (55.7% ± 4.7% and 57.1% ± 3.6%, respectively; *P* = 0.20 and *P* = 0.32, respectively) (Figs. 4a, 4b). HCFs cultured on Ti-(p)PMMA spread over a larger surface compared to being spread on PMMA, similar to casted Ti and TCPs. On untreated PMMA, cells tended to have a round shape and spread poorly (Fig. 5a). The alamarBlue testing of HCFs cultured on each surface demonstrated a gradual increase in relative fluorescence intensity as a function of incubation time, indicating cellular growth and proliferation over time on all experimental surfaces. HCFs seeded on Ti-(p)PMMA exhibited significantly higher metabolic activity compared to PMMA after 7 days of cell culture (*P* < 0.00001). HCFs cultured on Ti-(p)PMMA showed metabolic activity similar to HCFs on casted Ti and TCP controls (*P* = 0.14 and *P* = 0.13, respectively) (Fig. 5c).

**Figure 6.**
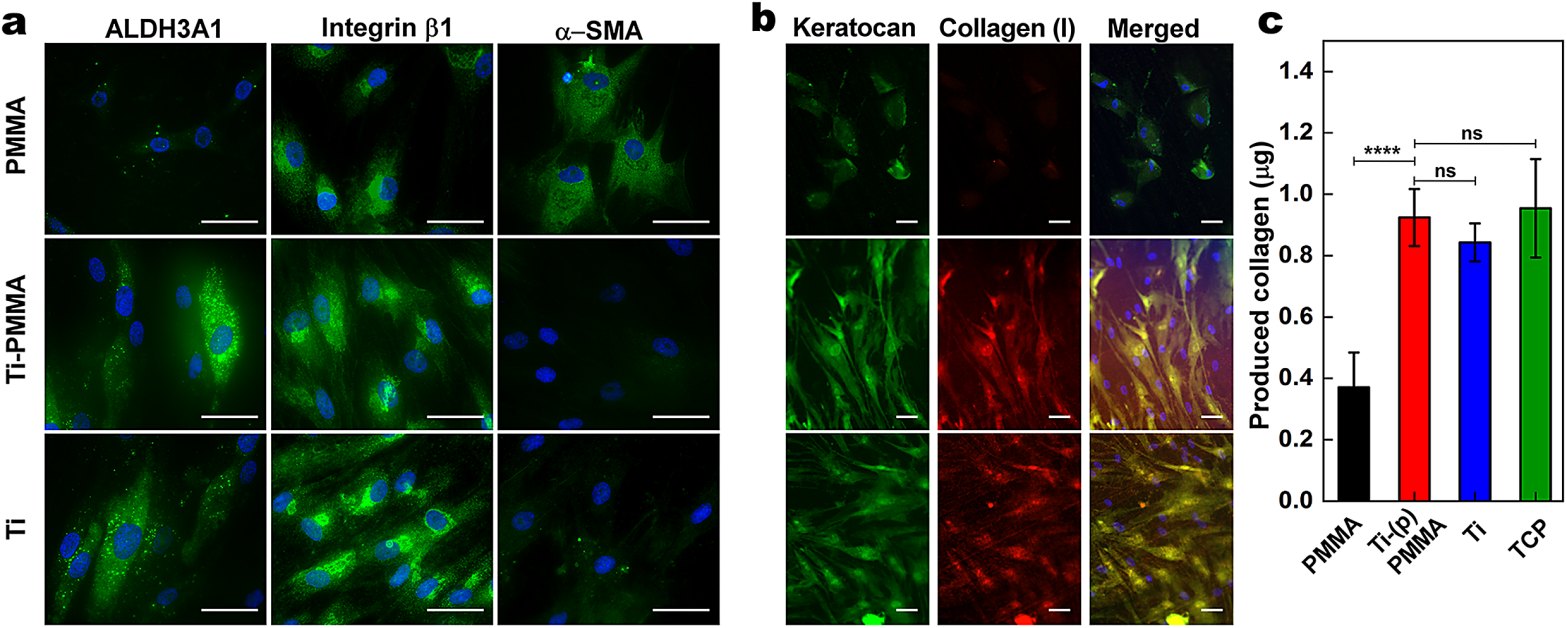
Representative fluorescent immunostained images of HCFs cultured on Ti-(p)PMMA compared to PMMA after incubation of 7 days (**a**) and 45 days (**b**). There were apparent differences in the expression of ALDH3A1 (*green*), integrin β1 (*green*), α-SMA (*green*), keratocan (*green*), and collagen I (*red*) on Ti-(p)PMMA and PMMA. All cell nuclei were counterstained using DAPI (*blue*). *Scale bar*: 50 µm. (**c**) Total collagen amount deposited on the specimens, as measured by hydroxyproline assay. ^*^P < 0.05, ^**^P < 0.01, ^***^P < 0.001, and ^****^P < 0.0001; ns, *P* > 0.05.

Further evaluation of the biological effects of Ti-(p)PMMA on cultured HCFs was performed using immunocytochemistry for ALDH3A1 (marker of keratocyte phenotype), integrin β1 (adhesion), and α-SMA (marker of fibroblast phenotype) (Figs. 6a, 6b) after 7 days of cell culture. All surfaces supported ALDH3A1 expression by cultured HCFs, but expression appeared relatively lower on PMMA. HCFs cultured on Ti-(p)PMMA strongly expressed integrin β1, whereas only a few cells cultured on control untreated PMMA showed such expression. HCF cells grown on Ti-(p)PMMA did not express α-SMA, in contrast to control PMMA where α-SMA expression was evident, the latter suggestive of a phenotypic switch from fibroblast to myofibroblast, as seen in proinflammatory and fibrotic responses.^37^

Immunocytochemistry analysis suggested that collagen I and keratocan deposition by HCFs cultured on Ti-(p)PMMA was higher (Fig. 6b) compared to on PMMA. In addition, extracellular matrix (ECM) deposited on Ti-(p)PMMA, casted Ti, and TCPs formed a visible membrane-like structure, covering the entire surface area of each sample, which was not observed with HCFs cultured on PMMA. The total amount of collagen on Ti-(p)PMMA (0.92 ± 0.09 µg/well) was similar to that for casted Ti (0.84 ± 0.06 µg/well) and TCPs (0.95 ± 0.16 µg/well) and was significantly higher than that observed with PMMA (0.37 ± 0.11 µg/well; *P* = 0.00024) (Fig. 6c).

**Figure 7.**
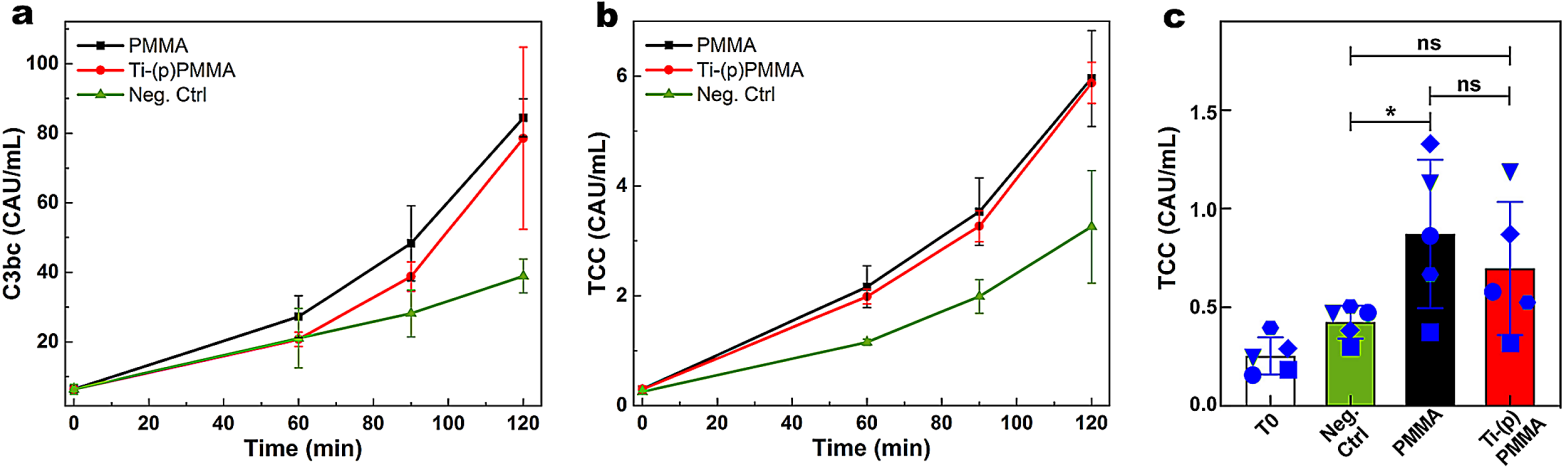
Activation of C3 and TCC in response to PMMA alone (*black*), Ti-(p)PMMA (*red*), and negative control (*green*) over time (60, 90, and 120 minutes); time zero min (T0) represents the status of complement activation immediately after drawing the blood from the donor). The levels of C3bc and TCC were detected, and the means of three observations were plotted against time (**a**, **b**). The increase of TCC level in response to both PMMA and Ti-(p)PMMA were again compared (*n* = 5) to each other and with negative control at 30 minutes; the distinct shape of the data points represents same donor (**c**). ^*^P < 0.05; ns, *P* > 0.05.

Complement activation by PMMA and Ti-(p)PMMA was assessed for two critical nodes in the complement activation system. Levels of C3bc and soluble TCC increased over time with exposure of whole blood (*n* = 3) to both PMMA and Ti-(p)PMMA (Figs. 7a, 7b). After 30 minutes of incubation with PMMA (*n* = 5), the level of TCC increased significantly (0.87 ± 0.38 CAU/mL) compared to the negative control (0.42 ± 0.08 CAU/mL; *P* = 0.003). Complement activation (as measured by TCC) by Ti (p)PMMA was not significantly different (0.69 ± 0.34) compared to the negative control (*P* = 0.29). There was no significant difference in TCC levels between PMMA and Ti-(p)PMMA (*P* = 0.64) (Fig. 7c).

## Discussion

The current study demonstrates that magnetron sputtering of Ti onto the stem of BKs is feasible and provides a mechanically robust and biocompatible surface. Our findings suggest that Ti sputtering on the BK stem may help corneal tissue adhesion and have the potential to improve clinical outcomes in patients with an implanted BK device.

Surface properties of medical implants have been shown to significantly impact subsequent cellular behavior and function.^24^^,^^38^ AFM studies have reported that plasma processing and Ti sputtering altered the topographical characteristics of PMMA and increased its roughness. The deposition of Ti led to the formation of plate-like structures, where the plates form junctions that protrude from the surface and create wall-type futures or edges that have greater heights compared to the center of the plates. Such features indicate the irregularity of the Ti film and are believed to originate from the low surface energy of PMMA compared to Ti combined with the intrinsic stress build-up during Ti thin-film growth, as reported by others.^39^^,^^40^ However, the final processed sample (Ti-(p)PMMA) was significantly smoother than casted Ti. Water contact angle studies also showed that Ti sputtering changed the wettability of the (p)PMMA surface and made it more hydrophilic. This is advantageous for biointegration, as it has been previously shown that moderately hydrophilic surfaces foster the highest levels of cell attachment.^41^ Our studies also demonstrate that plasma treatment and Ti sputtering of the PMMA stem can be accomplished without negatively impacting the optical properties of the device.

Coating of the PMMA surface must have sufficient mechanical stability to withstand cleaning, sterilization, and implantation of the device. Magnetron sputtered Ti film on PMMA has been explored previously,^36^ and it was shown that the plasma treatment significantly improved the bond strength between the sputtered film and underlying substrate.^42^ Our data show that the adhesion between a Ti sputtered film and plasma-treated PMMA (10.9 ± 1.1 MPa) is significantly higher than for non-treated PMMA (7.4 ± 1.1; *P* = 0.00002). For context, reported mean eyelid pressures based on three models of whole cell, imprint width, and Marx's line are 0.00051 ± 0.00001 MPa, 0.0011 ± 0.0004 MPa, and 0.0073 ± 0.0035 MPa, respectively, which are three to four orders of magnitude less than the adhesion strength of Ti-(p)PMMA.^43^ The increase in adhesion strength likely stems from (1) removal of organic and inorganic surface contaminants^42^; (2) removal of the so-called weak boundary layer made of oligomers present on the surface of polymer; (3) chemical modification of the surface, including oxidation of the surface and generation of carboxylic acid, along with generation of creation of surface dangling bonds, the latter being more reactive toward the incoming metal atoms^44^; (4) increased surface energy of the polymer to approach those of the sputtered metal; and (5) increased interface surface area.^45^ Mechanical evaluations also indicate that adhesion between the Ti film and underlying PMMA is not disrupted after incubation of Ti sputtered (p)PMMA in cell culture media, suggesting the stability of the coating under physiological conditions.

Scratch testing further demonstrated the stability of the sputtered Ti film and showed scratch resistance properties similar to those of casted Ti. In contrast, polydopamine,^46^ HAp over polydopamine,^17^ and TiO_2_/HAp dip-coated^19^ films have been shown to be relatively fragile with lower adhesion to PMMA, reducing the reliability and effectiveness of the coating and limiting potential applications for PMMA medical devices in clinical practice.^16^

Migration of corneal stromal cells from the host corneal tissue to the surface of the BK PMMA stem and subsequent proliferation and production of ECM are desirable for adhesion of corneal tissue to the stem in order to prevent gaps between the BK and the donor cornea that would otherwise permit ingress of microbes. Therefore, it is important to determine the biological interaction of HCFs with coated PMMA surfaces. It has been shown that Ti, especially with low roughness, displays enhanced biocompatibility with respect to corneal cells.^24^ Our studies have shown that HCFs cultured on Ti-(p)PMMA had cell confluency similar to those grown on casted Ti and TCP controls, the latter the gold standard for cell compatibility. Cell confluency was nearly threefold higher on Ti-(p)PMMA than on untreated PMMA, consistent with a salutatory effect of sputtered Ti. Moreover, HCFs seeded on Ti-(p)PMMA demonstrated almost twice as much metabolic activity as cells grown on untreated PMMA, as indicated by an alamarBlue assay. These data, along with characterizations of cell morphology and cell spreading, indicate that Ti sputtering on the surface of (p)PMMA enhances biocompatibility and cell viability.

Our study also found that HCFs seeded on a Ti-(p)PMMA surface expressed ALDH3A1, a keratocyte specific marker. In comparison, ALDH3A1 expression on untreated PMMA was reduced. This suggests that Ti-(p)PMMA is more permissive of a physiologically normal keratocyte phenotype.^47^ Keratocytes express numerous collagen-binding integrins (α2β1, α3β1, α5β1, and α6β1),^48^ which control corneal cell interactions with the surrounding ECM^49^ and play crucial roles in corneal health and disease. Moreover, integrins regulate mechanical stress and function as a bidirectional conduit for signals that originate inside and outside of cells.^50^ Our data indicate that integrin β1 was significantly upregulated when HCFs were plated on Ti-(p)PMMA as compared to PMMA, consistent with HCF adhesion to the Ti-(p)PMMA surface. In contrast, we did not observe α-SMA expression by HCFs cultured on Ti-(p)PMMA, whereas most HCFs cultured on control PMMA expressed α-SMA. Cell proliferation and differentiation are closely linked. Cell-cycle regulators and cell-type-specific gene expression work in conjunction for normal growth and development.^51^ Our studies suggest that PMMA surfaces, although not cytotoxic, do not support cell proliferation. Nonproliferative surfaces can induce ECM-related stress and lead to myofibroblast differentiation and α-SMA overexpression.^52^^,^^53^ This likely explains the overexpression of α-SMA on PMMA surfaces compared to Ti-(p)PMMA and is consistent with the suggestion that PMMA induces differentiation of HCFs to myofibroblasts, a process associated with inflammation and fibrosis.^37^ In contrast, we did not observe α-SMA expression by HCFs cultured on Ti-(p)PMMA, although most HCFs cultured on control PMMA expressed α-SMA. The expression of collagen I and keratocan (keratan sulfate containing proteoglycans), the most abundant corneal proteins (20% and 3.4% respectively),^54^^,^^55^ and ECM deposition overall were significantly higher on Ti-(p)PMMA compared to PMMA. HCFs lose their dendritic morphology and keratocan expression in high serum conditions. However, several studies have reported that the chemical, topological, and structural properties of the substrate can influence keratocan expression under in vitro conditions.^56^^,^^57^ Moreover, it has been shown that human keratocytes maintain their characteristic morphology and express keratocan when cultured on amniotic membrane even in the presence of high serum concentrations.^58^ This is in agreement with our study, which demonstrates the effect of surface properties on the expression on keratocan and α-SMA cell markers. Our results indicate that Ti-(p)PMMA promotes better cell adhesion, proliferation, and ECM deposition than PMMA and that the Ti sputtering made the substrate more biocompatible.

The complement system triggers inflammatory responses against a foreign body through a sequential enzyme cascade.^59^ As complement components function in the blood, the concept of complement-associated immune responses has been well studied for organs and tissues with a direct blood supply. The healthy cornea is avascular, but complement components make their way into the corneal stroma via the limbal vasculature, with a concentration gradient highest in the periphery of the cornea as compared to the center.^60^ In addition, in severe corneal disease conditions the avascular nature of the cornea is often compromised, leading to corneal neovascularization, with secondary increases in complement components in the stroma and direct contact of blood with corneal tissue, including any bioprosthetic implants. Moreover, it has been shown that depletion of complement components can improve the survival of corneal allografts.^61^ For this reason, it was important to determine whether Ti sputtering would induce increased complement activation relative to untreated PMMA. We tested complement activation by immersion of PMMA and Ti-(p)PMMA into whole blood. The complement activation product C3bc is generated when C3 is cleaved into C3a and C3b, which is then further cleaved to C3c and C3dg. TCC is formed when the C5b fragment binds to C6, C7, C8, and several C9 molecules (C5b-9). In the fluid phase, increased TCC is a highly reliable indicator of complement activation and humoral biocompatibility.^62^ In line with previously reported literature, we found that PMMA moderately activated the complement system.^63^ We demonstrated that Ti-(p)PMMA did not significantly increase complement activation compared to PMMA, which is widely used for indwelling medical devices, including intraocular lenses, and is generally considered biocompatible in the eye.

Corneal fibroblast interactions and behaviors (e.g., adhesion, proliferation, migration, ECM deposition) under in vitro conditions might not reflect those under in vivo conditions. The chemical composition, local biomechanics, nanoarchitecture of the surrounding matrix, spatial arrangement of cells with respect to their neighbors, and physiological communication between tissues and organs all orchestrate cell behavior under in vivo conditions, and these relationships are absent in vitro.^64^ In addition, long-term survival of a BK implant also relies on many other factors, including inflammatory and immune reactions to the implant, driven in part by the underlying disease process. Based on our study, we expect that the enhanced cellular adhesion, proliferation, spreading, and ECM synthesis on the Ti sputtered surface may lead to enhanced cell growth and ECM depostion in the interface between the native tissue and the implant, reduce exposure of the stroma to proinflammatory mediators and immune cells, and improve the clinical outcomes of the BK device. However, future studies in animal models are needed to determine the effect of the sputtered titanium surface on adjacent corneal tissue over time in vivo.

In conclusion, a Ti sputtered coating conferred favorable mechanical and biological properties to the BK PMMA stem. Ti sputtering of PMMA increased cell adhesion, proliferation, and collagen deposition and permitted maintenance of a more normal corneal stromal cell phenotype. These findings suggest that Ti sputtering may improve corneal tissue adherence to the BK stem and improve long-term clinical outcomes for recipients of the device.
